# Novel Class IIa-Selective Histone Deacetylase Inhibitors Discovered Using an *in Silico* Virtual Screening Approach

**DOI:** 10.1038/s41598-017-03417-1

**Published:** 2017-06-12

**Authors:** Kai-Cheng Hsu, Chang-Yi Liu, Tony Eight Lin, Jui-Hua Hsieh, Tzu-Ying Sung, Hui-Ju Tseng, Jinn-Moon Yang, Wei-Jan Huang

**Affiliations:** 1 Graduate Institute of Cancer Biology and Drug Discovery, College of Medical Science and Technology, Taipei Medical University, Taipei, Taiwan; 2 Graduate Institute of Pharmacognosy, Taipei Medical University, Taipei, Taiwan; 3 Kelly Government Solutions, Research Triangle Park, North Carolina, United States of America; 4 Institute of Bioinformatics and Systems Biology, National Chiao Tung University, Hsinchu, Taiwan; 5 Ph.D. Program in Biotechnology Research and Development, Taipei Medical University, Taipei, Taiwan; 6 Department of Biological Science and Technology, National Chiao Tung University, Hsinchu, Taiwan

## Abstract

Histone deacetylases (HDAC) contain eighteen isoforms that can be divided into four classes. Of these isoform enzymes, class IIa (containing HDAC4, 5, 7 and 9) target unique substrates, some of which are client proteins associated with epigenetic control. Class IIa HDACs are reportedly associated with some neuronal disorders, making HDACs therapeutic targets for treating neurodegenerative diseases. Additionally, some reported HDAC inhibitors contain hydroxamate moiety that chelates with zinc ion to become the cofactor of HDAC enzymes. However, the hydroxamate functional group is shown to cause undesirable effects and has poor pharmacokinetic profile. This study used *in silico* virtual screening methodology to identify several nonhydroxamate compounds, obtained from National Cancer Institute database, which potentially inhibited HDAC4. Comparisons of the enzyme inhibitory activity against a panel of HDAC isoforms revealed these compounds had strong inhibitory activity against class IIa HDACs, but weak inhibitory activity against class I HDACs. Further analysis revealed that a single residue affects the cavity size between class I and class IIa HDACs, thus contributing to the selectivity of HDAC inhibitors discovered in this study. The discovery of these inhibitors presents the possibility of developing new therapeutic treatments that can circumvent the problems seen in traditional hydroxamate-based drugs.

## Introduction

Epigenetic control has an important role in gene regulation through covalent chemical modifications of DNA, as well as by covalent post-translational modifications (PTMs) of histones^1^. One type of PTM, histone acetylation, is a reversible process that is regulated by histone acetyltransferases (HATs) and histone deacetylases (HDACs). Both HATs and HDACs target the lysine residues in the core histone tail. Negatively charged DNA binds tightly to positively charged histone that is characterized by the ε-amino group of the lysine residue. Acetylation of histone neutralizes the charge on the N^ε^-position of lysine residues, which converts the condensed heterochromatin into the relaxed euchromatin^2^. This unfolded euchromatin allows gene regulatory proteins and RNA polymerase to bind to DNA, leading to active DNA transcription. Thus, HDACs and HATs control the epigenetic process by regulating histone acetylation. In addition to histones, HDACs also regulate acetylation of some non-histone proteins such as tubulin, p21 and p53, which suggests that these enzymes are involved in certain cellular events as well^3, 4^. Studies reveal that HDACs are overexpressed in a broad spectrum of diseases such as inflammatory, neuron degenerative disorders and various cancer types^3, 5–7^. Therefore, HDAC inhibitors have potential applications in therapeutic treatment.

The mammalian HDACs can be divided into four classes depending on their sequence homology, sub-cellular distribution and catalytic activity. Class I, II and IV HDACs have the cofactor Zn^2+^ in the catalytic site and are regarded as classical zinc-dependent HDACs. In contrast, class III HDACs contain sirtuins 1–7 and are NAD^+^-dependent^8^. Class I (HDAC1, 2, 3 and 8) share the same homology as yeast Rpd3 and are preliminarily distributed in the nucleus of normal cells^9^. Class II HDACs can be subdivided into class IIa HDAC and class IIb HDAC. Class IIa enzymes (HDAC4, 5, 7 and 9) shuttle between the nucleus and cytoplasm and are located in specific tissues such as the brain, heart, and muscle. However, class IIb (HDAC6 and 10) are mainly located in the cytoplasm^9^. Class IV only contains HDAC11 and is located in the brain, heart, kidneys, skeletal muscle, and testis^9^.

The physiological role of HDAC isoforms has been elucidated by studies of knockout and transgenic mice and by RNA interference (RNAi)^10, 11^. Class I enzymes (HDAC1, 2, 3, 8) reportedly regulate differentiation, growth inhibition and apoptosis in cancer cells^12, 13^. Class IIb enzyme HDAC6 induces the acetylation of tubulin, microtubule^4, 14^ and HSP90^15^, which indicates that it is a potential target in the treatment of neurodegenerative diseases and cancers^16^. In contrast to class IIb HDAC enzymes, class IIa enzymes target unique client proteins as the substrates, which regulate the epigenetic process^17–21^. Class IIa enzymes are also relevant to some developmental and differentiation process^22^. Studies reveal that HDAC4 is a regulator of chondrocyte hypertrophy and has crucial roles in skeletogenesis and regulating neuronal cell death^23, 24^. Meanwhile, HDAC5 and 9 are known to induce cardiac hypertrophy and control heart function^25, 26^. Vascular integrity and regulation of T-cell development has been attributed to HDAC7^27, 28^. Recent studies reveal that class IIa HDACs are associated with the pathology of some neurodegenerative diseases including Alzheimer’s disease, Huntington’s disease, and mood disorders, which suggests that these enzymes should be targeted in treatment of CNS diseases^29–34^. Due to the conservative structure between individual HDACs, only few class IIa-selective HDAC inhibitors have been identified^35, 36^. Accordingly, the most important goals are developing agents that can selectively target class IIa enzymes and understanding the physiological role and clinical potential of such agents.

**Figure 1:**
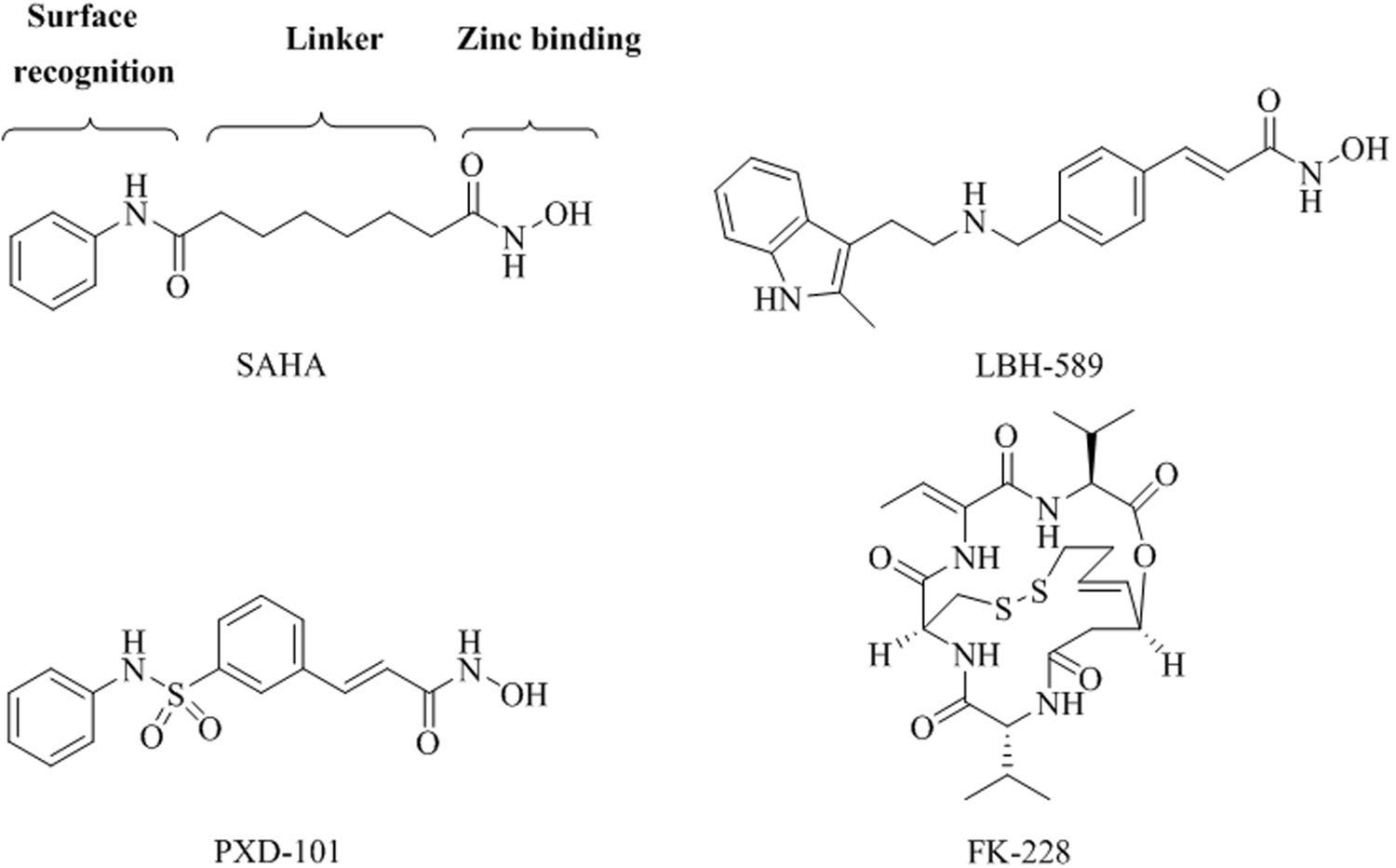
Chemical structures of FDA approved HDAC inhibitors.

The chemical structures of HDAC inhibitors generally contain three groups^35, 37^. These include the zinc binding group (ZBG) that includes hydroxamate, benzamide and thiol motifs that chelates the zinc ion at the bottom of the catalytic domain, a hydrophobic cap interacting with the surface rim of the enzyme that recognizes the HDAC isoform and a hydrophobic carbon chain linker that connects the zinc binding group with the cap and spans into the tunnel of the enzyme cavity. To date, several HDAC inhibitors approved by the FDA have been used to treat various cancer types. These compounds include suberoylanilide hydroxamic acid (SAHA)^38^, panobinostat (LBH589)^39^ and belinostat (PXD101)^40^ as well as FK228^41^ (Fig. 1). Except for FK228, most compounds are classified as hydroxamate compounds. The hydroxamate moiety in these inhibitors is commonly used as the ZBG because of its strong chelation ability. In addition to zinc ion, this moiety non-selectively chelates with other metal ions, including Mg^2+^, Mn^2+^, Fe^3+^, and Co^2+^, and acts as a cofactor for enzymes in cells as well^42^. This activity may induce various cellular side effects and undesirable outcomes. Additionally, hydroxamate-containing HDAC inhibitors usually have metabolic problems in glucuronidation and sulfation stages, which then lead to poor pharmacokinetic profiling^43, 44^. These problems, combined with the therapeutic potential of class IIa HDAC enzymes described above, motivate us to develop selective non-hydroxamate inhibitors of class IIa HDAC.

Protein Data Bank (PDB) is a publically available resource for three-dimensional structural data of large biological molecules^45^. Understanding 3D structures of proteins is essential for designing and optimizing compounds that bind to specific targets. Researchers can utilize PDB to obtain 3D structures of protein-ligand complexes and broaden our knowledge of how proteins interact with different chemical fragments and functional groups^46^. Therefore, PDB can also be harnessed to find new ZBGs, which is crucial for finding more specific HDAC inhibitors^35, 47^. In addition, MetLigDB, another publically accessible database, can be used to find structures that contain a variety of zinc binding groups and central metal ions in the active site of metalloproteins^48^.

Virtual screening techniques can identify ligands that have a high probability of binding to target proteins^49, 50^. This process includes ‘docking’ of small molecules to protein binding sites. Therefore, the structure of the target protein and the active or binding site must be identified. The National Cancer Institute (NCI) is a publically available database containing roughly 265,000 ligands for cancer research^51^. The large numbers of ligands and proteins found in the databases can be screened and ranked according to the most likely protein-ligand interaction. Top-ranked compounds can potentially lead to new therapeutic agents.

In our study, we utilized structure based virtual screening to find potential non-hydroxomate based inhibitors. Compounds with novel ZBG were identified and then ranked according to their docking scores. Experimental tests of high-ranked compounds on HDAC4 showed that they had a greater inhibitory effect when compared to SAHA. In addition, tests on class I and class IIb HDAC isozymes showed the inhibitors preference for class IIa. We further analyzed the interactions between the class I and class IIb HDAC isozymes and the inhibitors. The analysis of interactions between class I and class IIb HDAC isozymes and the inhibitors revealed a specific region of class IIa HDACs. The discovery of non-hydroxomate inhibitors may circumvent the disadvantages seen with hydroxamate based inhibitors.

## Results

### Overview of virtual screening strategy

**Figure 2:**
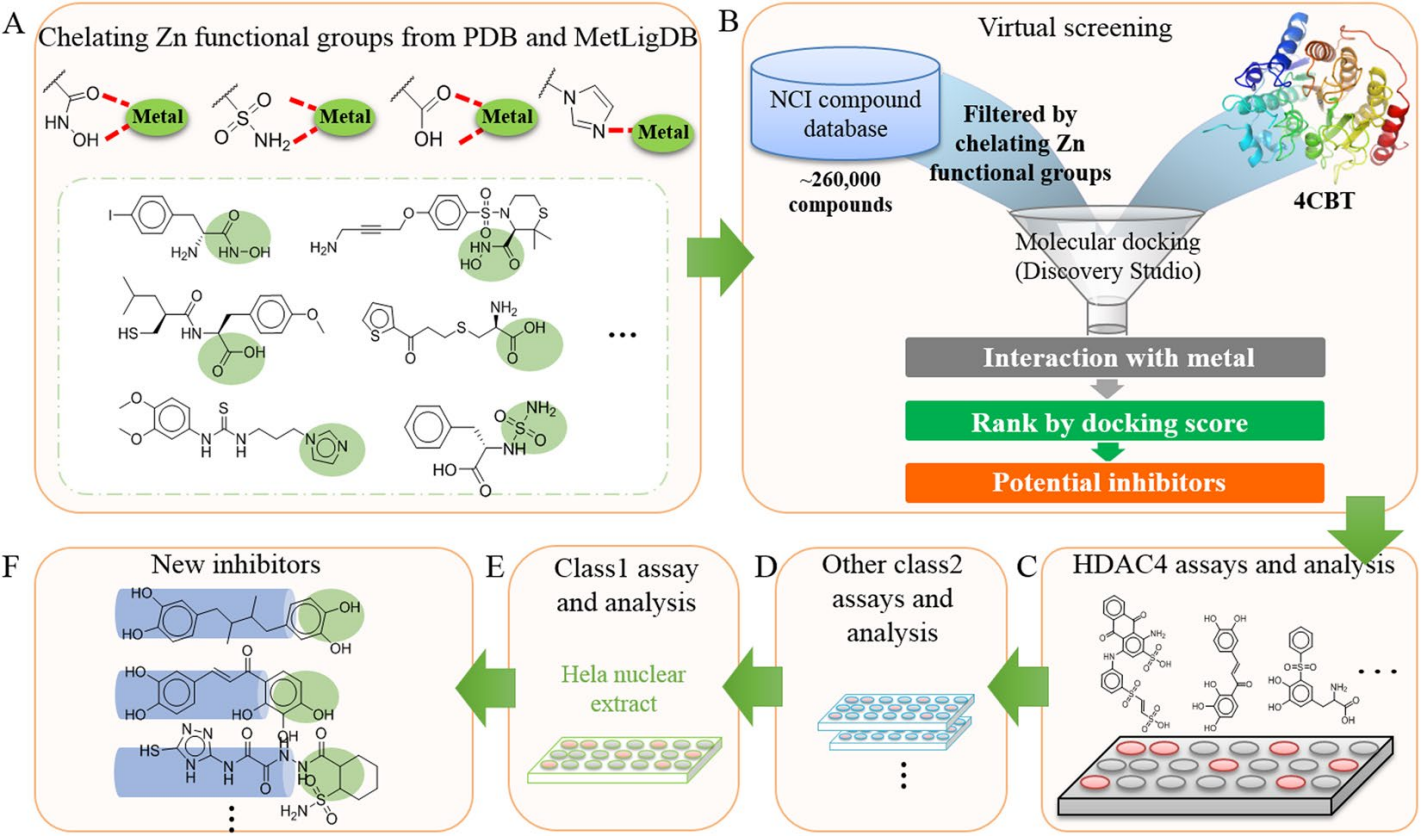
Framework of identifying non-hydroxamate inhibitors. (**A**) The structures of zinc binding compounds, which include hydroxamic acids, sulfonamides, carboxylic acids and imidazoles, are obtained from PDB and MetLigDB. The PDB and MetLigDB databases contains protein structures co-crystallized with metal binding ligands that can be used for novel inhibitor discovery (box with green dotted line, metal binding group located in green circle). (**B**) Structure-based virtual screening is performed by docking the NCI database compounds with the HDAC4 protein structure. The results are scored and ranked. The top 40 compounds were selected. (**C**) The candidate compounds undergo cellular assays to ensure docking results; the compounds with the strongest inhibitory effects were selected for further research. (**D**) The inhibitors were assayed against class IIa HDAC isozymes to determine inhibitory effects. (**E**) The inhibitors were assayed against class I HDAC to determine selectivity. (**F**) Discovered inhibitors with new ZBG that are selective for class IIa HDAC. Functional groups are indicated by green circle.

Figure 2 shows the framework of this study. First, various zinc binding groups were identified from PDB^45^ and Metalloprotein Ligand Interaction Database (MetLigDB)^48^, which provided clues for identifying new non-hydroxomate HDAC inhibitors. These groups include, but were not limited to, hydroxomic acid, sulfonamide, 1H-imidazole and carboxylic acid (Fig. 2A). Compounds were selected from the NCI^52^ database (approximately 265,242 compounds) that contained functional groups that interacts with the zinc ion. This initial screening yielded 2,890 compounds with the above functional groups. These structures were then docked and ranked by their binding energy score in BIOVIA Discovery Studio (DS) (Fig. 2B)^53^. Next, the potential inhibitors were tested *in vitro* for HDAC4 inhibition (Fig. 2C). Further tests of class IIa isozymes, such as HDAC5, HDAC7, HDAC9 and HeLa nuclear extract containing class I HDACs were performed to determine inhibitor selectivity (Fig. 2D–F). These assay results revealed new non-hydroxomate inhibitors that are selective for class IIa HDACs.

### Selecting Compounds with Metal Binding Groups from PDB and MetLigDB

**Figure 3:**
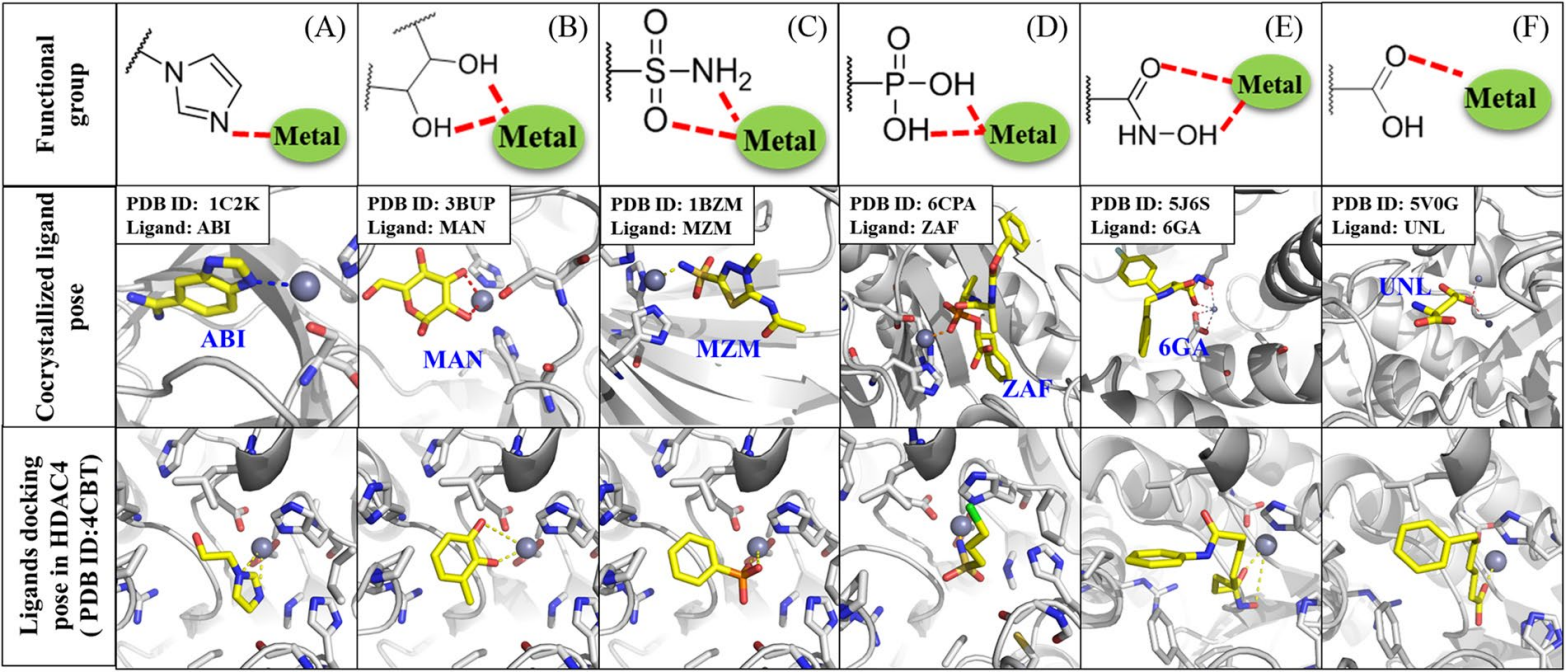
Validation of metal binding groups in HDAC. Structure of metal binding group for imidazole (**A**), diols (**B**), sulfonamids (**C**), phosphonates (**D**), hydroxamic acids (**E**), and carboxylic acids (**F**) located at top. The middle panel show structures of co-crystallized ligands with functional groups. The PDB ID of ligands are shown in blue. The small fragment docking docked in HDAC4 crystal structure is shown at the bottom. Grey sphere represents zinc ion.

The ligand-protein co-crystallized structures of many inhibitors with metal binding groups can be found on PDB. In addition, MetLigDB, which provides a list of zinc binding groups. Observing and analyzing known metal binding groups provide clues for designing non-hydroxomate inhibitors. Figure 3 shows examples of co-crystallized compounds with different metal binding groups obtained from PDB. These structures include imidazoles, diols, sulfonamids, phosphonates, hydroxamic acids, and carboxylic acids. Small fragment structures of similar metal binding groups were obtained and docked with HDAC4 using the CDOCKER tool in DS. The docking results showed that the groups can form interactions with the zinc ion (bottom panels, Fig. 3). Our docking results reduced the 265,242 compounds found on the NCI database to 2,890 candidate compounds that contained at least one of the metal binding functional groups.

### Structure-Based Screening with NCI Database

The program CDOCKER in DS was used to prepare HDAC4 (PDB ID: 4CBT) and to dock the candidate compounds. The 2,890 candidate compounds were ranked by their docking scores. Compounds that did not form metal interactions were removed. The docking results were then examined with knowledge-based analyses in order to eliminate false positive ligands. Specifically, compounds that formed at least one key interaction with HDAC4 residues F812, H842, F871, and L943^29^ were selected. Finally, 40 available candidates were requested from NCI (Supplementary Fig. 1).

### Assay of HDAC4 Enzymatic Activity

**Figure 4:**
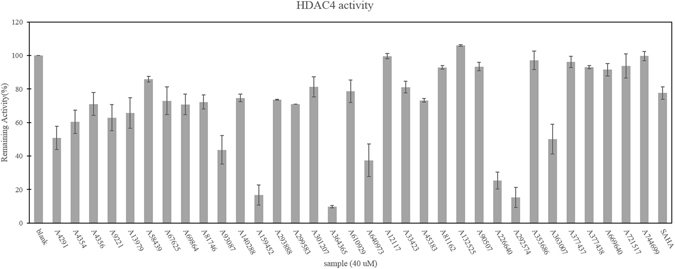
HDAC4 inhibitory activities of 33 candidate compounds. The HDAC4 inhibitory activity was measured with HDAC fluorometric activity assays. The 33 candidate compounds and SAHA were tested at a concentration of 40 µM.

A fluorogenic based assay^54^ was used to validate the 40 candidate compounds. Seven compounds were excluded from the assay due to their poor solubility in DMSO. The remaining 33 compounds were initially screened for HDAC4-inhibiting activity at a concentration of 40 μM using SAHA as the reference compound^55^. Of these compounds, eight compounds designated A4291, A93087, A159452, A226640, A363007, A364365, A640973 and A292574 had significantly higher inhibitory activities against HDAC4 compared to SAHA (Fig. 4).

Next, the HDAC4 IC_50_ values were determined for the eight compounds. Compounds A159452 (IC_50_ = 4.47 µM), A363007 (IC_50_ = 4.16 µM) and A364365 (IC_50_ = 2.58 µM) exhibited much higher activity compared to SAHA (IC_50_ > 40 µM) (Table 1). Notably, compound A364365 had an approximately 20-fold higher inhibitory effect compared to SAHA. Furthermore, the inhibitory activity of compounds A4291 and A640973 were moderate compared to SAHA. Despite the high inhibitory activities of compounds A93087 and A292574 in the preliminary screening of HDAC4 inhibition (Fig. 4), further assays were unable to produce an IC_50_ value for these two compounds. This may be due to the compounds’ degradation within the buffer.

**Table 1:** Structures of six compounds and SAHA and their IC_50_ values (µM) of HDAC4 inhibitory activity.

Compound	Group	IC_50_	Structure
A4291	Catechol	24.7	
A159452	Catechol	4.4	
A226640	Sulfone	14.9	
A640973	Sulfone	17.4	
A364365	Sulfone	2.5	
A363007	Phosphonic acid	4.1	
SAHA	Hydroxamic acid	>40	

### Ligand-protein interaction analysis of HDAC4

**Figure 5:**
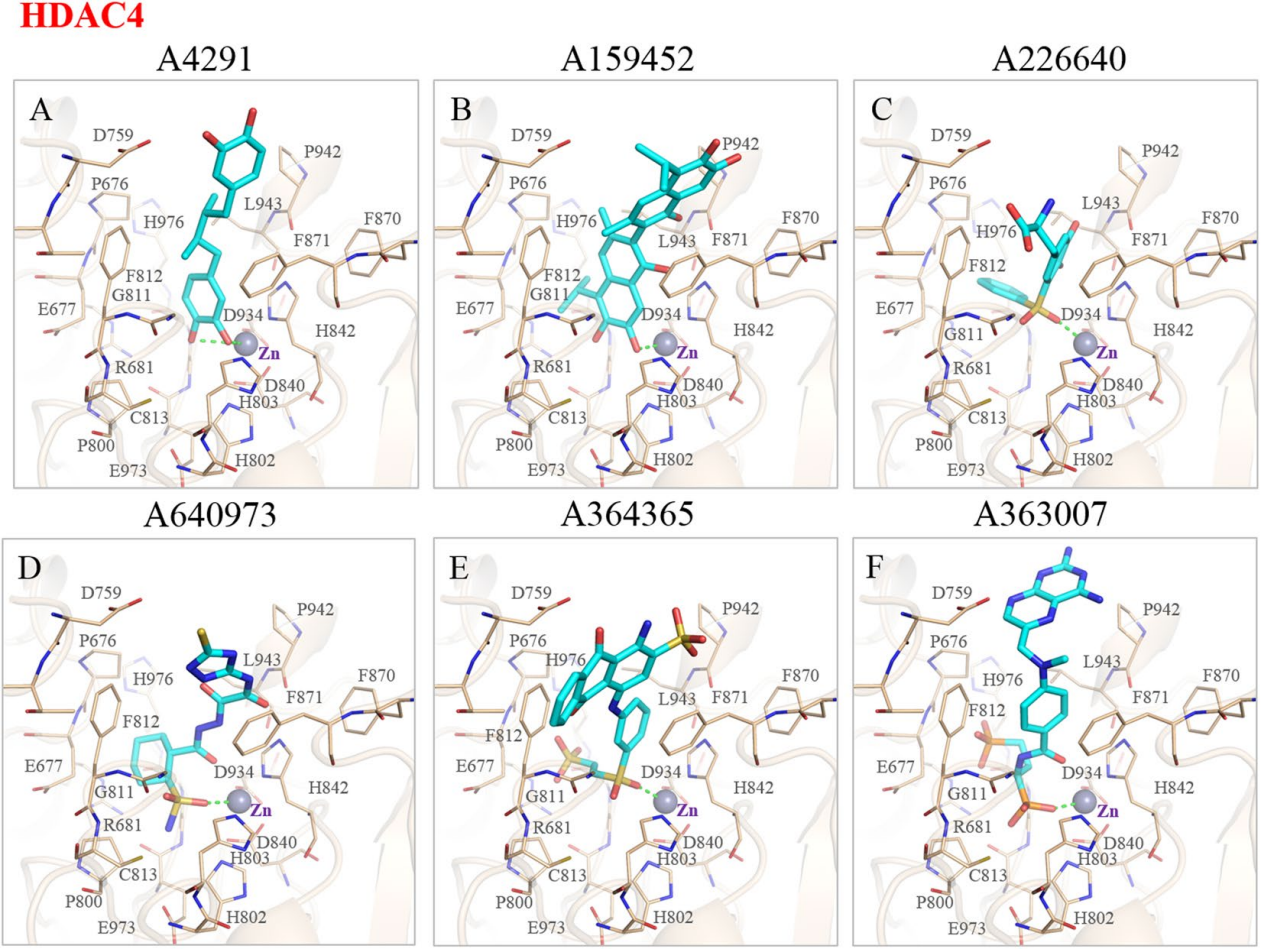
Interaction analysis of six potential inhibitors in HDAC4 binding site. The docking poses of six inhibitors in the binding site of HDAC4 (**A**–**F**) reveals the spatial conformations within the active site. Atoms are colored by type whereas lines (tan) represent HDAC4 protein residues. The zinc ion (grey sphere) is located within the active site. Amino acid residues are listed as shown.

To understand interactions mechanisms between the inhibitors and HDAC4, we performed an interaction analysis using the BIOVIA DS^53^ platform. Along with 3D stick models (Fig. 5), 2D diagrams were created to visualize inhibitor interactions within HDAC4 (Supplementary Fig. 2A–G). A heat map of residue interactions was created to determine consensus residues (Supplementary Fig. 2H). Inhibitors A4291 and A159452 are composed of similar moieties and interactions. However, A4291 lacks the two inner aromatic rings and does not make hydrophobic contacts with P676 (Fig. 5A). Inhibitor A4291 and A159452 forms hydrophobic interactions with F812, H842 and F871 and L943 (Supplementary Fig. 2A,B). In addition, inhibitor A159452 has more hydrophobic interactions with amino acids residues (Supplementary Fig. 2H). Inhibitor A159452 has two phenyl rings and two catechol rings at both terminals (Fig. 5B). One of the terminal hydroxyl groups on the catechol rings coordinates to the catalytic zinc ion near the bottom of the binding cavity of HDAC4, while the other forms a hydrogen bond with G974. This compound is also able to form hydrophobic contacts with hydrophobic residues, such as P676, F812, H842, F871, and L943 due to its two aromatic ring structures at both terminals. This creates a narrow groove for the fragment to fit in HDAC4’s center. It would appear that the extra aromatic ring is key to forming more interactions for HDAC4 when compared to inhibitor A4291 (Table 1, Supplementary Fig. 2A,B).

The inhibitors A226640, A640973, and A364365 possess a sulfonyl group that forms coordinated bonds with the zinc ion of HDAC4. Compound A226640 contains two phenyl rings, which are joined by a sulfonyl group that forms a coordinated bond with the zinc ion. Hydrogen bonds with P676 and H803 and G975 are formed to anchor the inhibitor within the cavity (Fig. 5C). Additionally, van der Waals interactions occur between aromatic residues H842 and F871 (Supplementary Fig. 2C).

A640973 can be subdivided into a 1,2,4-triazolidine-3-thiol moiety at one terminal, forming a hydrogen bond with D759 and a cyclohexane at the other terminal with an amino(hydroxyl) methylsulfanium group attached to one of the carbons on the cyclohexane ring (Fig. 5D). The two terminals are connected by an aliphatic carbon chain composed of nitrogen atoms and protruding oxygen atoms, one of which forms a hydrogen bond with the polar amino acid H842. The nonpolar and aromatic residues P676 and F871 further restricts A640973 rotation by sandwiching the compound and forming hydrophobic interactions (Fig. 5D, Supplementary Fig. 2D). Compound A364365 begins with an anthraquinone structure with occasional protruding nitrogen and sulfonyl groups, with the oxygen forming hydrogen bonds with F812 and G975 (Fig. 5E). The head of the structure is followed by another single phenyl ring that ends with two sulfonyl groups that form a coordinated bond with the catalytic zinc ion. As F871 is in the same plane of alignment with the short aliphatic link between the aromatic rings of the inhibitor, the chemical structure is partially held in place by hydrophobic interactions. In addition, aromatic residues H842 and F812 sandwiched the inhibitor within the active site (Supplementary Fig. 2E).

Finally, compound A363007 is unique to the rest of the inhibitors in that it contains a phosphoric acid moiety to form coordinated bond with the catalytic zinc ion. This compound starts with a pteridine-2,4-diamino connected by a carbon. It contains a tertiary amine that connects to a single aromatic ring with an aliphatic chain that extends with two phosphoric acid moieties in the horizontal plane. These two phosphoric acids are essential for forming hydrogen bonds with its surrounding residues, which include D759, H802, G811 and G975 (Fig. 5F). Like A226640, both aromatic residues H842 and F871 and the hydrophobic residue P942 are involved in the formation of van der Waals contacts within the catalytic site (Supplementary Fig. 2F). Importantly, these inhibitors formed interactions with lower pocket residues F812, H842, F871, and L943^29^ (Supplementary Fig. 2G).

### Comparisons of the Selectivity of Inhibitors in Targeting Class IIa HDAC Isozymes and HDAC Isozymes

Table 2 reveal the inhibitory activities of the identified inhibitors against a panel of HDAC isoforms that include class IIa (HDAC5, 7 and 9), class IIb (HDAC6), and HeLa nuclear HDACs that mostly contain class I (HDAC1, 2, and 3). The identified inhibitors have lower inhibitory activity against class IIb and HeLa nuclear HDACs compared to SAHA. However, inhibitors A159452, A363007, and A364365 show proficient inhibitory activity for HDAC4, HDAC5, and HDAC9 (Table 2) In contrast to its weak activity against HDAC4, 5, and 7, inhibitor A4291 was the strongest inhibitor against HDAC9. This suggests inhibitor A4291 is a HDAC9 isoform-selective inhibitor (Table 2). Inhibitor A226640 displayed selectivity for HDAC5 over other class IIa enzymes, whereas compound A640973 had inhibitory activity for HDAC5 and HDAC9 (Table 2). These experimental results indicate that the identified inhibitors target HDAC isoforms within class IIa enzymes and can thus be recognized as class IIa-selective HDAC inhibitors.

**Table 2:** The IC_50_ (µM)^a^ values for the inhibition of selected compounds against class IIa, class IIb and HeLa nuclear HDACs.

Compound	Class IIa				Class IIb	Hela Nuclear HDACs
	HDAC4	HDAC5	HDAC7	HDAC9	HDAC6	
A4291	24.74	23.45	>40	0.58	>40	>40
A159452	4.47	3.35	>40	7.14	>40	>40
A226640	14.90	2.94	>40	12.88	>40	>40
A640973	17.48	2.92	>40	2.46	>40	>40
A364365	2.58	1.33	14.59	2.38	>40	>40
A363007	4.16	1.27	16.16	7.84	>40	>40
SAHA	>40	>40	>40	>40	0.010	0.04

^a^Each values are presented as the mean of three independent experiments.

### Analysis of Ligand-Protein Interaction Between Class IIa HDAC Isozymes

We further analyzed the six inhibitor interactions with HDAC5, HDAC7 (PDB ID: 3ZNS) and HDAC9. Because the crystal structure of HDAC5 and HDAC9 is unavailable, a homology model was created and used instead^56^. Our cellular analysis of the six inhibitors yielded inhibitory effects for HDC4, HDAC5 and HDAC9 (Table 2). However, their effectiveness was reduced with HDAC7 isozyme. This suggests a possibly disparity with the structure of the active site. The stick model for HDAC5, HDAC7 and HDAC9 can be seen in supplementary Fig. 3–5 for analysis.

**Figure 6:**
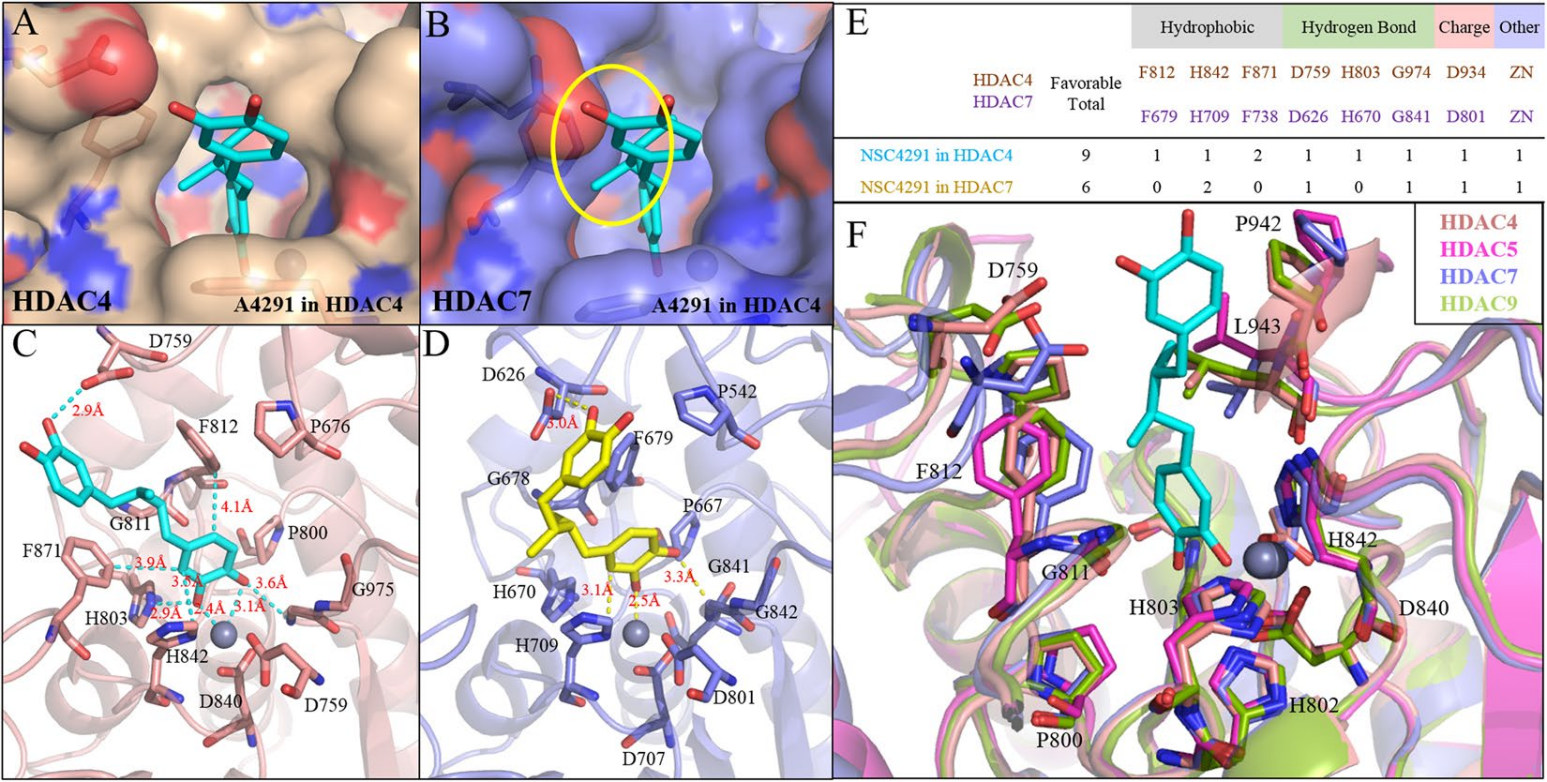
Comparison between HDAC4 and HDAC7 active sites. The surface model of HDAC4 (**A**) and HDAC7 (**B**) binding site and include docking structure of inhibitor A4291 (blue) docked from HDAC4. Yellow circle indicates the position of aspartate amino acid found in HDAC7. (**C**) The stick model of HDAC4 (red) active site is docked with inhibitor A4291 (blue). (**D**) The stick model of HDAC7 (purple) active site is docked with the inhibitor A4291 (yellow). (**E**) The residues of HDAC4 and HDAC7 active site reveal contrasting bond favorability for inhibitor A4291. The numbers in the table represent the number of interactions at corresponding amino acid residues. (**F**) All class IIa structures are aligned to reveal conserved residues within the active site. The residue number corresponds to HDAC4. Note that the aspartic acid residue of HDAC7 is lower compared to the class IIa isozymes.

Our structural analysis of HDAC7 revealed a cavity opening that is roughly 2 Å smaller compared to HDAC4 (Fig. 6A,B). When compound A4291, posed from HDAC4, is transferred to HDAC7, the smaller cavity size sterically hinders its ability to interact within the active site. This difference is attributed to the position of D759 on HDAC4, which is located on a higher plane compared to D626 on HDAC7 (Fig. 6A,B). Furthermore, the stick model shows how inhibitor A4291 can enter HDAC4 and form the crucial zinc bond (Fig. 6C). When inhibitor A4291 was docked into HDAC7, we observed that the aromatic rings are oriented closer together compared to their positions in HDAC4 (Fig. 6D). The distances between the bonds within the cavity site is smaller in HDAC4 than in HDAC7 (Fig. 6C and 6D). Moreover, the inhibitor makes less interactions with key residues in HDAC7 compared to HDAC4 (Fig. 6E). Superimposing class IIa HDACs reveal many conserved residues within active site (Fig. 6F). Additionally, the aspartic acid for HDAC5 and HDAC9 is located on a higher plane compared to HDAC7, which reduces the cavity surface and increases steric hindrance (Fig. 6F).

### Structural Differences Between Class I and Class IIa HDAC Determines Inhibitor Selectivity

**Figure 7:**
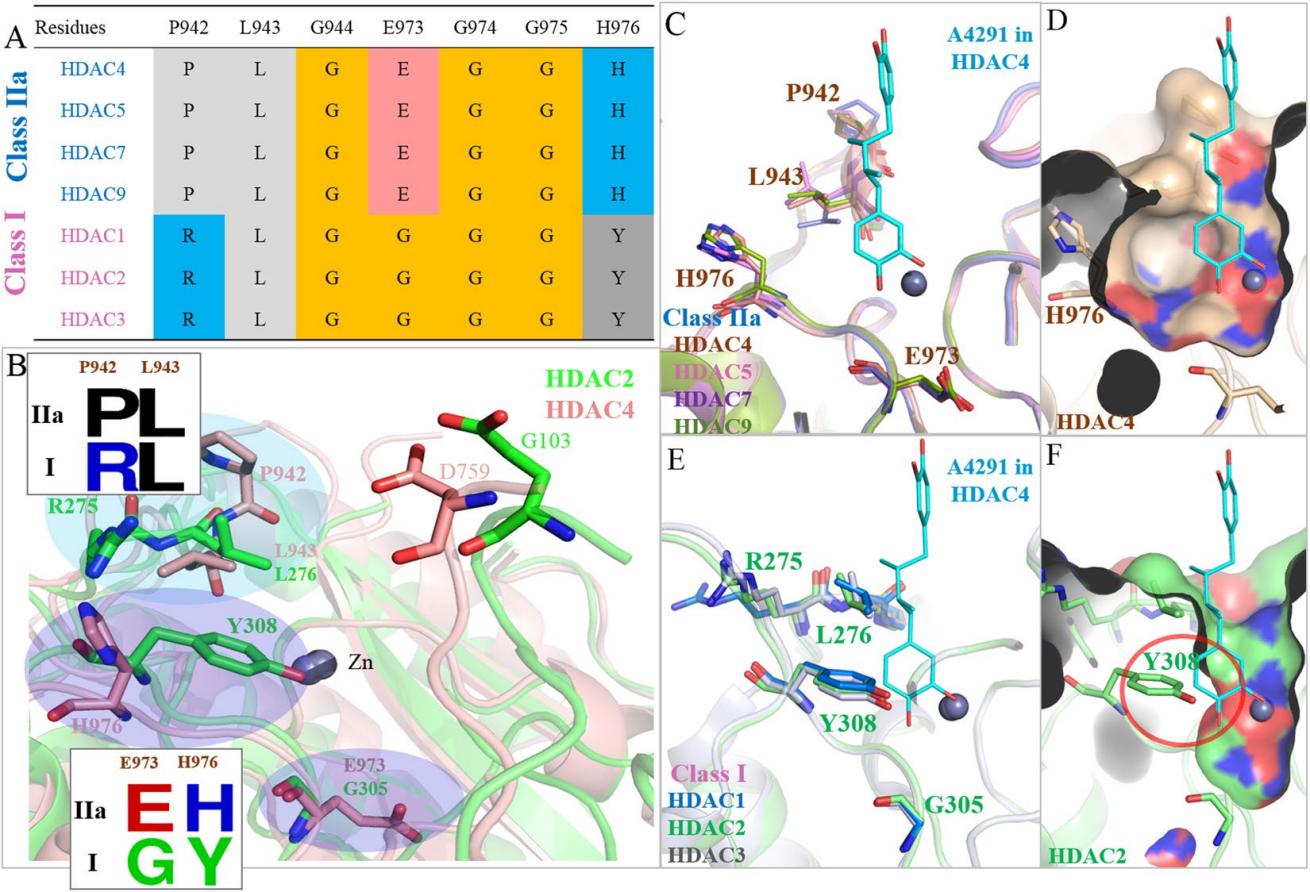
Comparison of Class I and Class IIa HDAC cavities. (**A**) Sequence alignment of residues within the catalytic site of class I (pink) and class IIa (blue) HDAC isozymes as shown. Residue numbering for HDAC4 is located on top. (**B**) Structural alignment of HDAC2 (green) and HDAC4 (brown) with different residues (boxes) are shown at designated positions. Blue circles highlight residue differences. (**C**) Stick model of class IIa isozymes are aligned with inhibitor A4291 (blue) docked from HDAC4. (**D**) The surface model of the HDAC4 active site is superimposed with inhibitor A4291 to show the relative cavity size. (**E**) Stick model of class I isozymes aligned with inhibitor A4291 (blue) docked from HDAC4. Enzymes are labeled as shown. (**F**) The surface model of HDAC2 (green stick model) is superimposed with inhibitor A4291 docked from HDAC4 (blue). Location of residue Y308 is indicated by red circle. Note the reduced space within the active site due to Y308. Zinc is indicated by grey sphere.

Inhibitor selectivity was further investigated between class I, IIa, and IIb HDACs. These structures were aligned using the Jalview^57^ multiple sequence alignment tool (Supplementary Fig. 6). The positions of differing residues within the active site of class IIa were chosen and compared. Residues P942, E973 and H976 of HDAC4 are conserved among class IIa, whereas class I had arginine, glycine and tyrosine residues at these positions, respectively (Fig. 7A). The structures of HDAC2 and HDAC4, class I and class IIa respectively, were aligned to compare residue locations (Fig. 7B). Class I and IIa differ between a tyrosine and histidine residue, respectively. Previous studies revealed a HDAC4 histidine to tyrosine mutation at this location increases catalytic deacetylation activity to the level of class I HDACs^29, 58^. A stick and surface model revealed a large cavity within class IIa HDACs (Fig. 7C,D). Class I HDACs tyrosine residue contains an aromatic ring that extends into and reduces the cavity site (Fig. 7E,F).

We further analyzed if the tyrosine to histidine change is responsible for the SAHA inhibition activity. The sequence alignment reveals class IIa residues containing a histidine, while class I and IIb contain a tyrosine residue (Supplementary Fig. 6). We aligned HDAC2 (PDB ID: 4LXZ) and HDAC6 (PDB ID: 5EEI), class I and IIb respectively, that contained a crystallized SAHA structure (Supplementary Fig. 7A). The interaction analysis show tyrosine Y308 (HDAC2) and Y745 (HDAC6) providing a hydrogen donor to form a hydrogen bond with the SAHA carbonyl group. This stabilizes the binding of SAHA within the enzyme. Inhibitor A4291, docked from HDAC4, was aligned into class I, IIa and IIb cavity site. For class I and IIb, the tyrosine residue is conserved. This reduces the cavity size, due to tyrosine’s aromatic ring extending towards the zinc ion. In contrast, class IIa HDACs contain conserved histidine residue, which is situated distantly to the zinc ion (Supplementary Fig. 7B).

**Figure 8:**
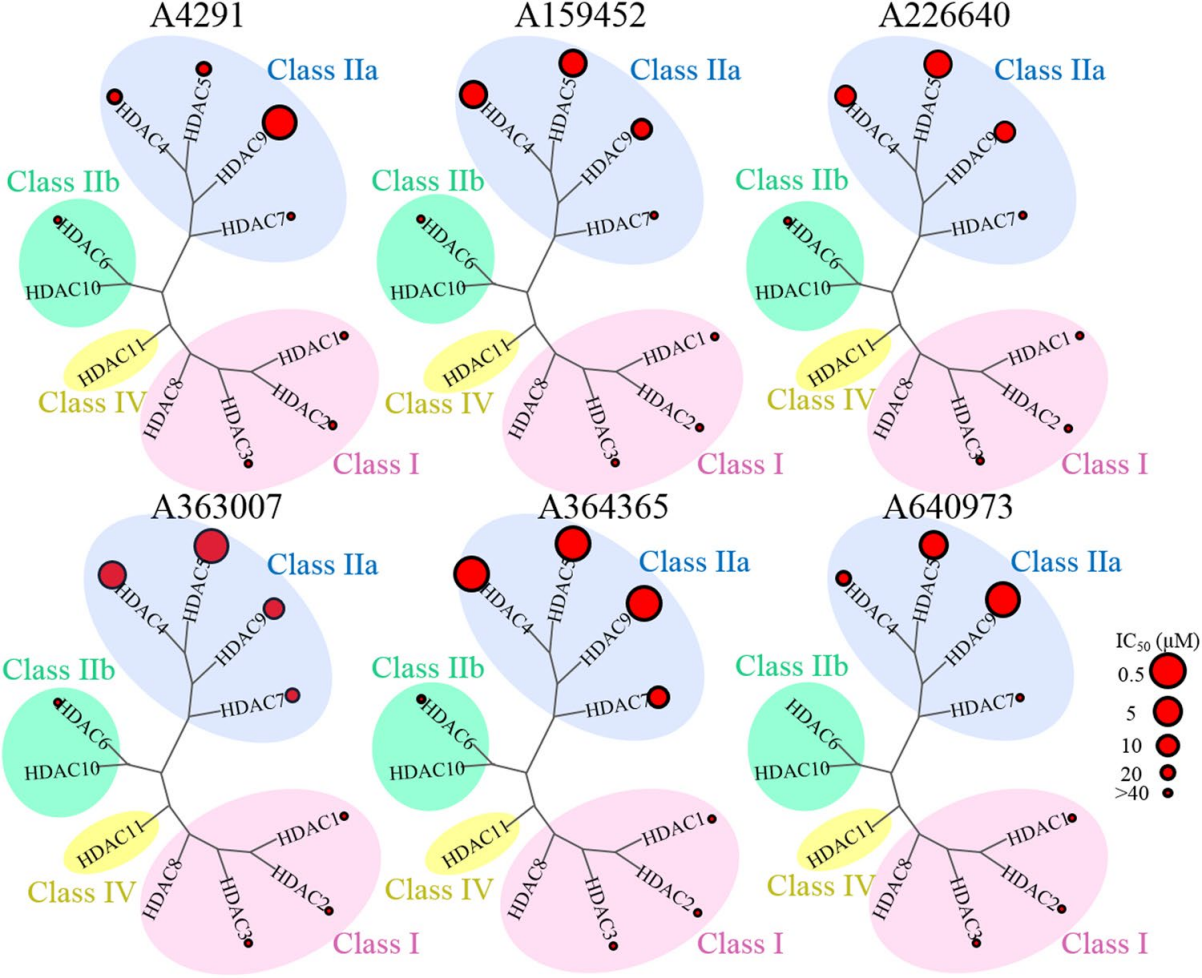
Selectivity of new inhibitors for HDAC isozymes. The phylogenetic tree of HDAC isozymes, class I, class IIa, class IIb and class IV HDAC are grouped according to sequence homology and shaded as pink, blue, green and yellow, respectively. The red circles represent IC_50_ values as observed in Table 2, with the largest circle indicating the lowest IC_50_ values.

Further tests against class IIb HDAC6 isozyme and class I HDACs revealed IC_50_ values greater than 40 µM, with only SAHA, which was used as a control, having a low IC_50_ value (Table 2). A phylogenetic tree of the HDAC isozymes are grouped according to sequence homology and revealed that the identified inhibitors are more effective against class IIa HDACs (Fig. 8). This further confirms the identified inhibitors’ preference towards class IIa HDACs. Importantly, the identified inhibitors are able to form key interactions within the lower pocket of the catalytic site and coordinates with the zinc ion. Our experimental results revealed non-hydroxamate based inhibitors with unique ZBG that are selective for class IIa HDACs, which can be used as possible lead compounds for future drug development.

## Discussion

The structure-based virtual screening used in this study discovered new non-hydroxamate inhibitors that are selective towards class IIa HDACs. Most HDAC inhibitors have potent activities through metal chelation with the hydroxamate, which can form strong interactions with zinc ion. However, the hydroxamate moiety will non-selectively target a variety of metal ions. This strong, but unspecific, metal binding activity will bring about unfavorable side effects^37, 55, 59^. One way to circumvent these shortcomings is to improve the zinc binding of HDAC inhibitors, or to design non-hydroxamate based inhibitors^60–62^. Unfortunately, most non-hydroxamate ZBGs are less potent than their hydroxamate counterparts^55^. Therefore, discovery of new, effective and selective ZBG for class IIa HDAC inhibitors have important therapeutic applications.

This study used the PDB and MetLigDB databases to obtain structures of available proteins and ligands. Our experimental results revealed six new compounds displaying strong inhibition of HDAC4, HDAC5 and HDAC9. Because the study primarily used BIOVIA Discovery Studio^53^ to screen the candidate compounds, false positive may impact the ranking scores. To reduce any false positives, we further docked our 40 candidate compounds into HDAC4 using LeadIT^63^ and iGEMDOCK^64^ software. Overall, results from three programs produced similar compound ranking (Supplementary Fig. 8A). For example, A363007 was ranked at 1 across the three programs. However, inhibitor A159452 ranks were more varied. The consensus scores, which averaged the ranking obtained by the three programs, revealed an enhancement for our compound rankings (Supplementary Fig. 8B). The results suggest that using different methods can help the ranking process. While some discrepancy is present between the DS and the consensus rankings, the HDAC fluorogenic assay was able to filter any false positives observed using DS.

Interaction analysis of the six compounds in HDAC4, the homology models HDAC5 and HDAC9, and HDAC7 revealed the important moieties and interactions within the active site (Fig. 5, Supplementary Figs 3–5). Due to the conserved ZBG, researchers generally assume similar behaviors between HDAC class IIa isozymes^65^. In general, we observed a larger opening in the catalytic opening of HDAC7 compared to HDAC4. Specifically, the D626 position in HDAC7 was on a lower plane compared to residue D759 of HDAC4 (Fig. 6). This was not observed with HDAC5 and HDAC9. The D626 of HDAC7 would prevent inhibitors from forming the necessary interactions within the active site. Thus, the identified inhibitors received a higher IC_50_ value with HDAC7 (Table 2). Additionally, the IC_50_ values of the identified inhibitors revealed their preferences for Class IIa HDACs (Fig. 7), which is significant due to class IIa HDACs association with various cancers and diseases^66–68^. This is in stark contrast to most of the current HDAC inhibitors, which are pan-inhibitory^28, 37, 69^.

Class I and class IIa HDACs differ in their deacetylase activity, due to a tyrosine to histidine change within their active site^29, 58^. Our interaction analysis revealed that the conserved tyrosine residue of class I reduces its cavity size and disrupts the biding ability of the identified inhibitors (Fig. 7). Thus, the identified inhibitors are specific for class IIa HDAC isozymes (Fig. 8). In addition, SAHA is a strong inhibitor for class I and IIb HDACs. However, SAHA has been shown to inhibit class IIa at concentrations that are not clinically significant^70^. The histidine, rather than the tyrosine, in class IIa is a key residue in determining their deacetylase activity^29, 58^. Besides the histidine change, the active site of class IIa HDACs are conserved across other HDAC isozymes. In fact, a histidine to tyrosine mutation will significantly increase deacetylase activity of class IIa HDACs^17^. With class IIa HDACs involvement in various diseases^29–34^, understanding their structural differences presents a first step in developing an effective therapeutic treatment.

## Conclusion

We used structure-based virtual screening to identify six novel non-hydroxamate inhibitors that target HDACs, which are involved in a variety of diseases including various cancers, neurodegenerative diseases and mood disorders. This makes HDACs a target with great therapeutic potential. These six compounds effectively inhibited class IIa HDAC activity, showing a selectivity not observed in most HDACi. To our knowledge, these findings add inhibitors with novel ZBG and offer a wide range of choices for developing inhibitors that specifically target class IIa HDAC isozymes.

## Method

### Compound selection with specific functional groups

Compound selection was performed by BOVIA Pipeline Pilot (http://accelrys.com/products/collaborative-science/biovia-pipeline-pilot/) with the 2D function graphical script. Individual chemical structures of hydroxamic acids, bezenediols, sulfonamides, bisphosphonates and other zinc binding groups were prepared as a SMART file using SMARTSeditor (https://www.biosolveit.de/SMARTStools/). The components, SMARTS Reader and Substructure Filter From Tag, were then used to select NCI compounds that possess the groups.

### Molecular docking of classified NCI compounds with HDAC 4 crystal structures

The CDOCKER program in DS^53^ was used to dock the compounds to the binding sites. The 2,890 compounds were prepared by the automatic ligand preparation function in DS Small Molecules Tools for generating coordinates in 3D dimension and fixing bad valences. Input receptor was defined as the structure of HDAC4 (PDB ID: 4CBT) X-ray structure prepared by the automatic ligand preparation function in DS Macromolecules Tools to build the missing loop. The protonated state was adjusted to 7.4 pH value, with the binding site defined by the co-crystallized ligand compound in a 10.2 Å radius sphere. The calculations and scoring were performed with the Docking Optimization function of CDOCKER using the default setting parameters. In the docking experiment, each ligand generated 10 random conformations with 1,000 dynamic steps in target temperature of 1,000 K and 10 orientation and refined with 800 maximum bad orientations. The CHARMm forcefields and Momany-Rone ligand partial charge method was employed for the scoring function. The calculated results were saved with the top 10 hit poses based on the scores of –COCKER ENERGY (negative of the energy) in one file and then examined in the 3DGraphic panel of DS. Structure for HDAC4 (PDB ID: 4CBT) and HDAC7 (PDB ID: 3ZNS) was obtained from PDB.

The compounds were ranked by their energy score. The top 20% ranked compounds were selected with the following criteria: 1) compounds that contained functional groups that create a coordinated bond with a distance within 2.4 Å from the zinc ion, 2) compounds that form interactions with at least one of the key HDAC4 residues F812, H842, F871, and L943^29^, which make up a hydrophobic channel, and 3) the availability of the compounds. The selected compounds were examined using the 3D graphics of DS. Finally, we selected 40 compounds.

After enzymatic assays, the selected compounds were ranked using LeadIT^63^ and iGEMDOCK^64^ to reduce possible false negatives obtained from DS. The 40 candidate inhibitors were docked into HDAC4 (PDB ID: 4CBT) and ranked by their energy scores according to each program. The data set contained six positive (identified inhibitors) and 27 false positives (candidate compounds). Seven compounds were removed, due to their insolubility in DMSO. The true positive hit rate defined as *I*/*T* (%), where *I* is the number of the identified inhibitors among the *T* highest-ranking compounds. The consensus scores were obtained by averaging the scores from each program. The hit rate was graphed to visually observe and compare the data set between programs.

### Histone Deacetylase Activity Assay

*In vitro* HDAC inhibition was measured by the HDAC fluorometric activity assays^71^. Enzymes, inhibitors, and substrates were diluted with HDAC buffer (15 mM Tris·HCl pH 8.1, 0.25 mM EDTA, 250 mM NaCl, 10% v/v glycerol). Briefly, 10 μL of diluted HDACs such as HeLa nuclear extract, HDAC4, HDAC5, HDAC6, HDAC7, AND HDAC9 were added into each well of the 96-well microtiter plate (Black immunoplate, SPL Life Sciences Co., Ltd.). The tested compounds (10 μL) were added to the assay buffer ( < 1% DMSO) at different concentrations diluted with 40 μL HDAC buffer then pre- incubated at 30 °C for 5 min. The enzymatic reactions were started with an addition of 40 μL fluorogenic substrates such as Boc-Lys(Ac)-AMC 10 μM for HeLa nuclear extract and HDAC6, Boc-Lys(TFA)-AMC^72^ 10 μM for HDAC4, fluorogenic HDAC class IIa substrate, Catalog #: 50040 (BPS Bioscience, San Diego, CA) at 10 μM for HDAC5, HDAC7 and HDAC9 into the HDAC buffer. After incubation and brief shaking at 37 °C for 30 min, the reactions were terminated by addition of 100 μL trypsin solution (10 mg/mL trypsin in 50 mM Tris·HCl pH 8, 100 mM NaCl, 2 mM SAHA). After additional 20 min incubation, fluorescence was measured (extinction and emission wavelengths at 355 nm and 460 nm) with a VICTOR X2 Multilabel Plate Reader (PerkinElmer). The fluorescence in wells without test compounds (0.1% DMSO, negative control) was set as 100% enzymatic activity and the fluorescence in wells with enzyme eliminated was set as 0% enzymatic activity. The fluorescence ratio of test compounds to negative control was defined as percentage of remaining enzyme activity. The IC_50_ values were calculated by linear regression of the data.

### Homology modeling

Amino acid sequences for HDAC5 and HDAC9 were obtained from the GenBank^73^ database and used as targets for homology modeling using the SWISS-MODEL server (https://swissmodel.expasy.org)^74^. The server performed a target-template sequence alignment after searching X-ray template proteins in PDB to generate 3D models for all target sequences.

### Data Preparation, Statistics, and Bioinformatics

The protein interactions were analyzed and visualized using BIOVIA DS^53^ and PyMOL^75^ software. The heat map was generated using the Broad Institute web-based tool Morpheus (https://software.broadinstitute.org/morpheus/). The files were uploaded and formatted to expected column headings as described in input file documentation. Custom colors were selected to represent high sequence identity (red) to low sequence identity (pink) as displayed in Supplementary Fig. 2. The 2D interaction figures were produced in BIOVIA DS. The multiple sequence analysis was performed with Jalview^57^ using the webserver TCoffee with default settings. The alignment used the Clustal X color scheme.

## Electronic supplementary material

Supporting information
