# Supplementary material for: Novel Class IIa-Selective Histone Deacetylase Inhibitors Discovered Using an in Silico Virtual Screening Approach
Source: Sci Rep. 2017 Jun 12;7:3228. doi: 10.1038/s41598-017-03417-1 (PMC5468338; doi:10.1038/s41598-017-03417-1)
Supplement: Supplementary file 1 — Supporting information [file 41598_2017_3417_MOESM1_ESM.pdf]

Supporting Information

Novel Class IIa-Selective Histone Deacetylase Inhibitors Discovered Using an *in Silico* Virtual Screening Approach

Kai-Cheng Hsu[1], Chang-Yi Liu[2], Tony Eight Lin[1], Jui-Hua Hsieh[3], Tzu-Ying Sung[4], Hui-Ju Tseng[5], Jinn-Moon Yang[4,6] and Wei-Jan Huang[2,7*]

[1]Graduate Institute of Cancer Biology and Drug Discovery, College of Medical Science and Technology, Taipei Medical University, Taipei, Taiwan
[2]Graduate Institute of Pharmacognosy, Taipei Medical University, Taipei, Taiwan
[3]Kelly Government Solutions, Research Triangle Park, North Carolina, United States of America
[4]Institute of Bioinformatics and Systems Biology, National Chiao Tung University, Hsinchu, Taiwan
[5]Ph.D. Program in Biotechnology Research and Development, Taipei Medical University, Taipei, Taiwan
[6]Department of Biological Science and Technology, National Chiao Tung University, Hsinchu, Taiwan
[7]Ph.D Program in Biotechnology Research and Development, College of Pharmacy, Taipei Medical University

*Correspondence should be addressed to:
Dr. Wei-Jan Huang, Graduate Institute of Pharmacognosy, Taipei Medical University, No.250, Wu-hsing St., Taipei 11031, Taiwan; Tel: +886-2-27361661 ext. 6152; Fax: +886-2-27355276; E-mail: wjhuang@tmu.edu.tw

Analysis of ligand-protein interaction in HDAC5 and HDAC7

We compared the six inhibitor interactions with HDAC5 and HDAC7 (PDB ID: 3ZNS) to understand their different activities. Because the crystal structure of HDAC5 is unavailable, a homology model was used instead. Our analysis of the six compounds showed that they yield similar interactions with HDAC5 and HDAC4 (Table 2). A4291 (Supplementary Fig. 3A) and A159452 (Supplementary Fig. 3B) share similar moieties and binding activities. Their cyclohexane-1,2-diol moieties form coordinated bonds with the zinc ion, as well as hydrogen bond with the polar amino acid residue H833. Van der Waals interactions are formed with the hydrophobic L973 amino acid residue. Inhibitors A226640 (Supplementary Fig. 3C), A640973 (Supplementary Fig. 3D) and A364365 (Supplementary Fig. 3E) contain sulfonyl moieties that create coordinated bonds with the zinc ion. They also share interactions with residues H872 and G1004. Inhibitor 363007 (Supplementary Fig. 3F) contains a phosphoric moiety and forms hydrogen bonds to R710 and G1004, but hydrophobic bonds to H872 and F900.

Our IC_{50} results showed the identified inhibitors having less inhibitory effect on HDAC7 (Table 2). Inhibitors A4291 (Supplementary Fig. 4A), A159452 (Supplementary Fig. 4B), A226640 (Supplementary Fig. 4C) and A640973 (Supplementary Fig. 4D) were unable to form interactions with the zinc ion within the catalytic site. In contrast, Table 2 displayed inhibitors A364365 (Supplementary Fig. 4E) and A363007 (Supplementary Fig. 4F) with weak inhibitory effects.

Finally, an interaction analysis was performed on HDAC9. The structure of HDAC9 is unavailable; therefore, a homology model was developed based on HDAC4. The interaction analysis of HDAC9 showed similar results to HDAC4 and HDAC5 (Supplementary Fig. 5A-F).

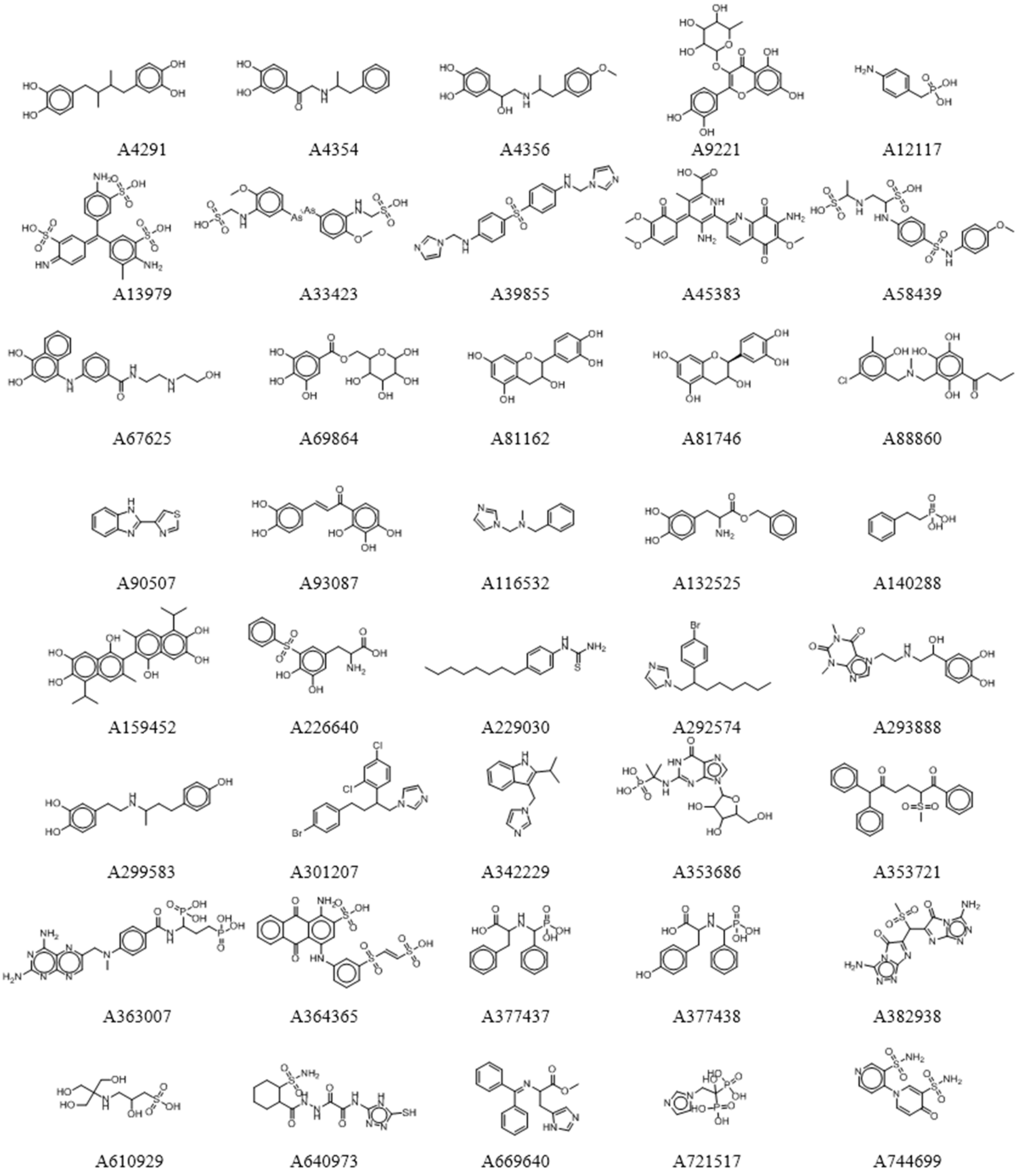

Supplementary Figure 1. The structures of 40 candidate compounds.

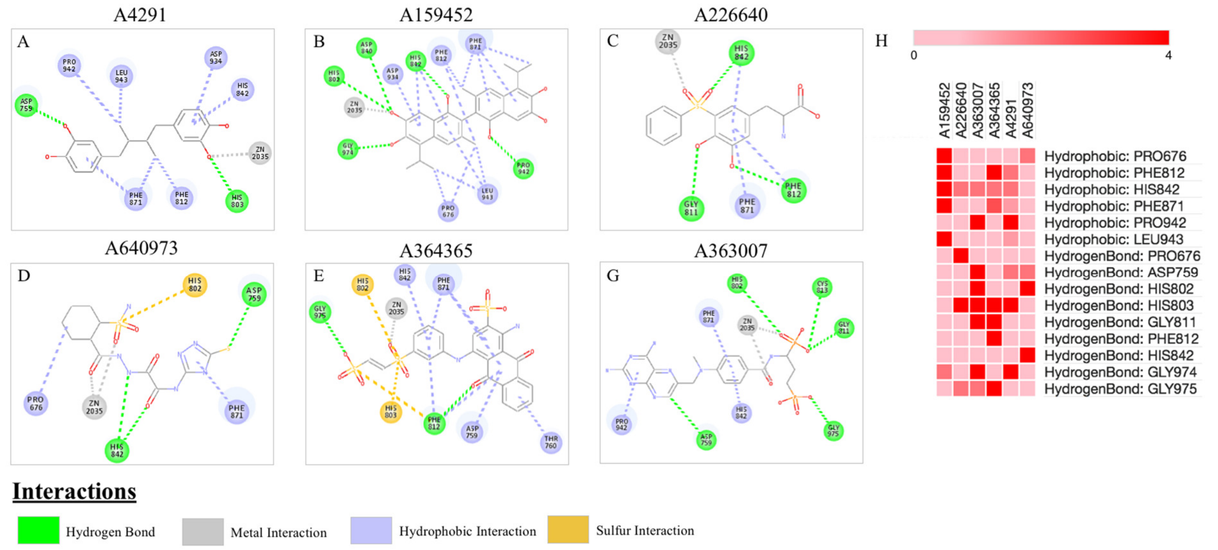

Supplementary Figure 2. Residue interactions of identified inhibitors.

(A-F) 2D diagram interactions between the identified inhibitors and cavity site residues of HDAC4 as developed from DS. Residues and bonds are colored by interaction types. (G) The heat map of interactions between cavity site residues and HDAC4. The highest interactions (4) are shaded red, whereas the lowest interactions (1) are shaded pink.

HDAC5

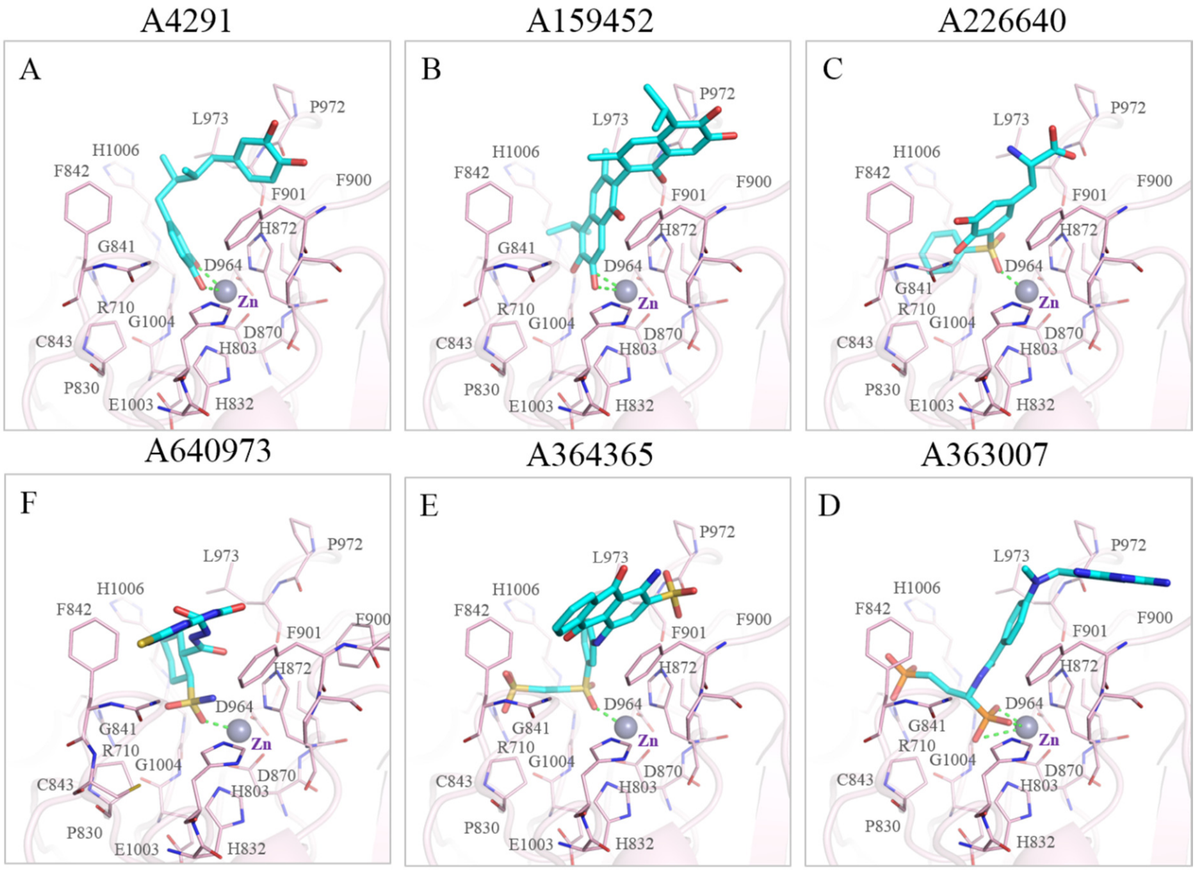

Supplementary Figure 3. Interaction analysis between identified inhibitors and HDAC5. The docking poses of six inhibitors in the binding site of HDAC5 (A-F) reveals the spatial conformations within the active site. Atoms were colored by type whereas lines (pink) represent HDAC5 protein residues. The zinc ion (grey sphere) is located within the active site, with dashed line indicating coordinate binding. Amino acid residues are listed as shown.

HDAC7

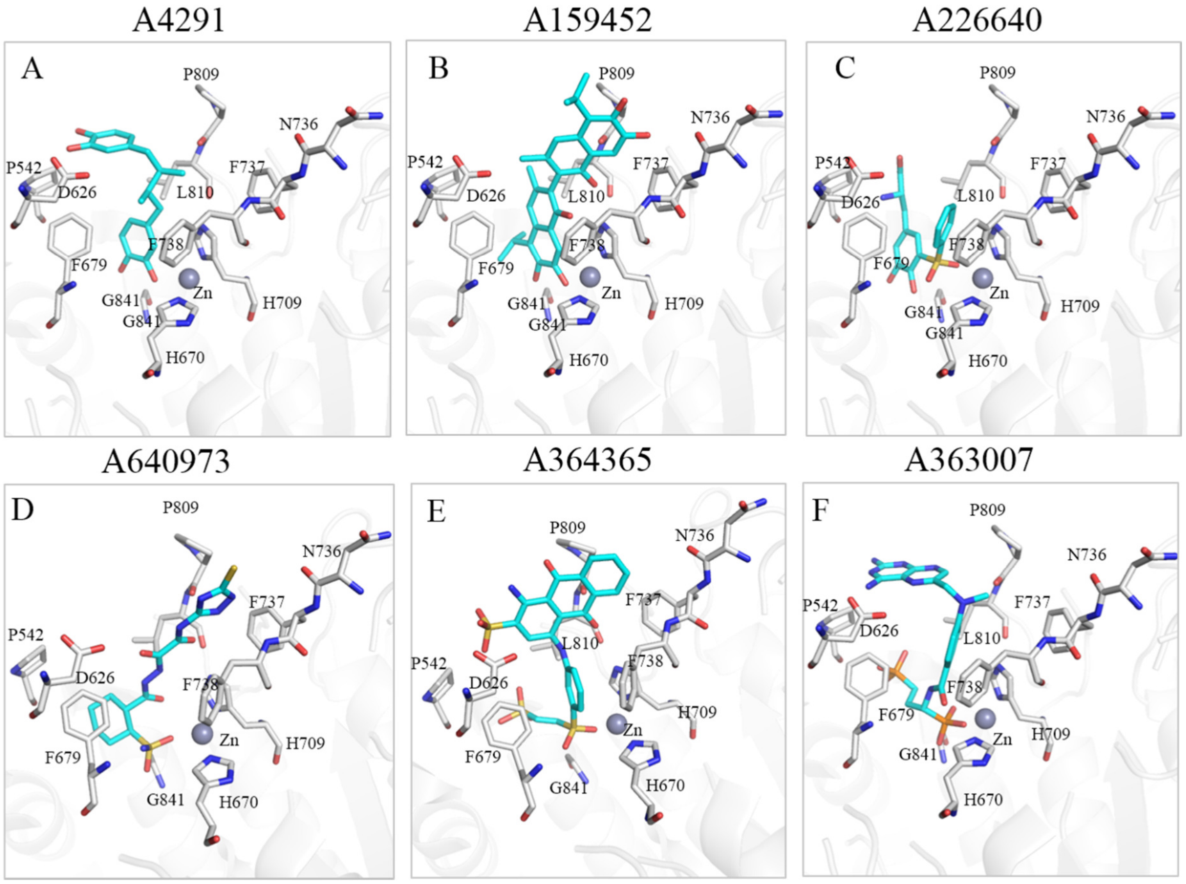

Supplementary Figure 4. Interaction analysis between identified inhibitors and HDAC7. The docking poses of six inhibitors in the binding site of HDAC7 (A-F) reveals the spatial conformations within the active site. Atoms were colored by type whereas lines (grey) represent HDAC7 protein residues. The zinc ion (grey sphere) is located within the active site, with dashed line indicating coordinate binding. Amino acid residues are listed as shown.

HDAC9

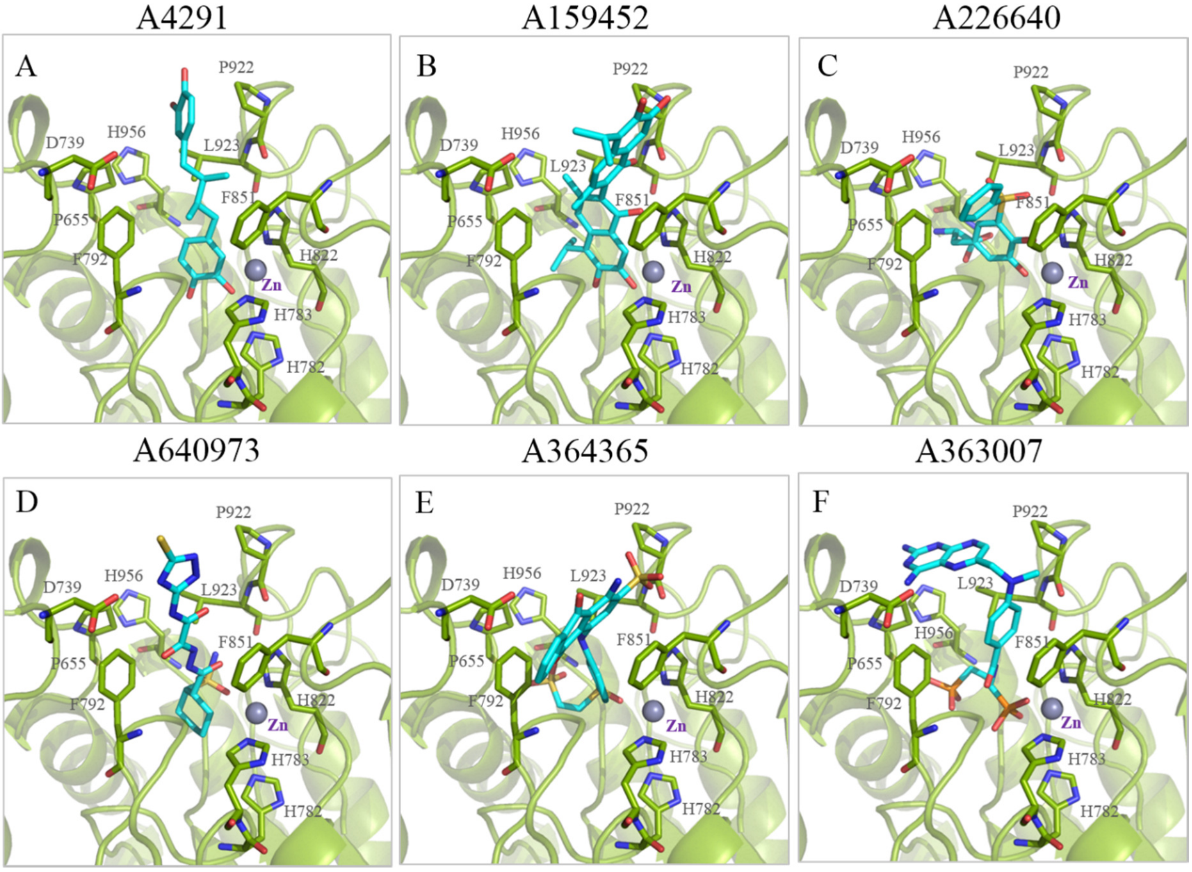

Supplementary Figure 5. Interaction analysis between identified inhibitors and HDAC9. The docking poses of six inhibitors in the binding site of HDAC9 (A-F) reveals the spatial conformations within the active site. Atoms were colored by type whereas lines (green) represent HDAC9 protein residues. The zinc ion (grey sphere) is located within the active site, with dashed line indicating coordinate binding. Amino acid residues are listed as shown.

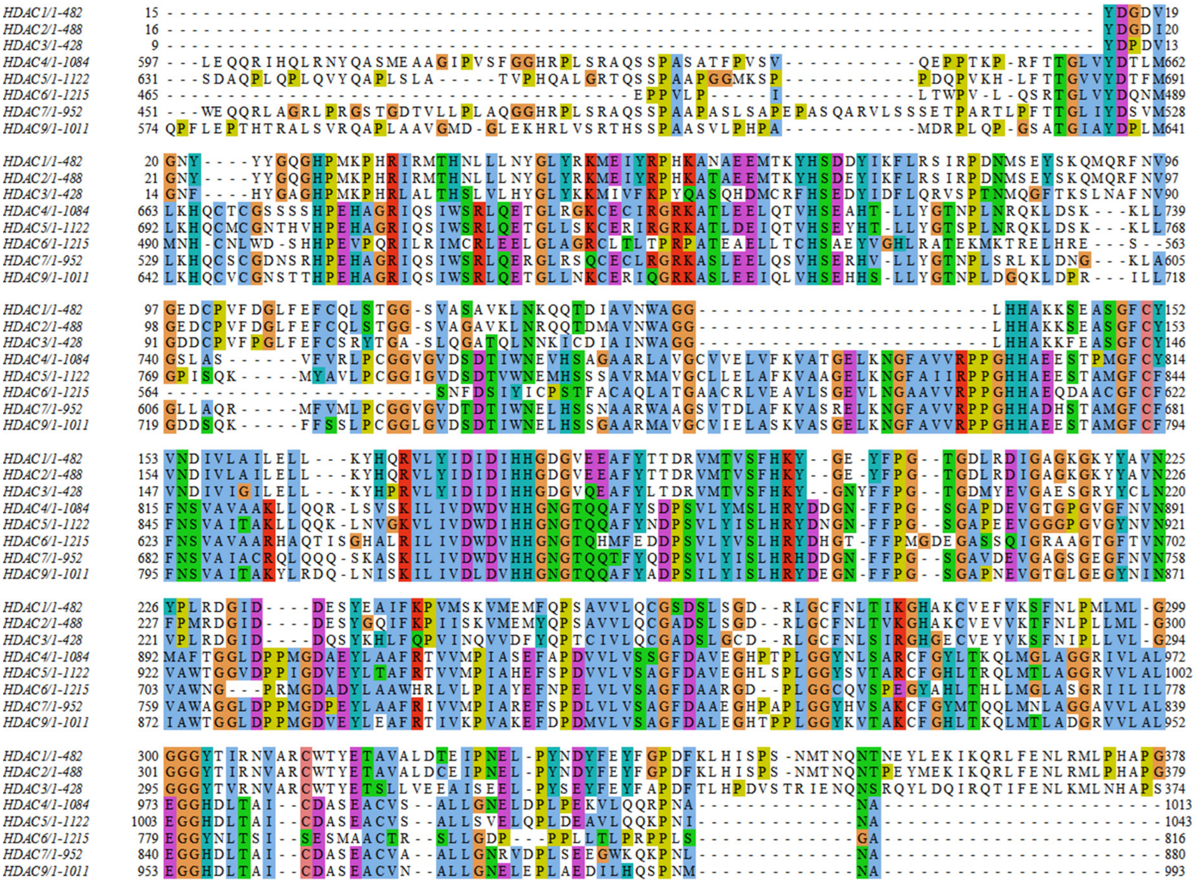

Supplementary Figure 6. Multiple sequence alignment of HDAC isozymes.

The HDAC isozyme sequences was obtained and aligned. Amino acid residues are color coded and highlighted to show residue consensus at numbered position.

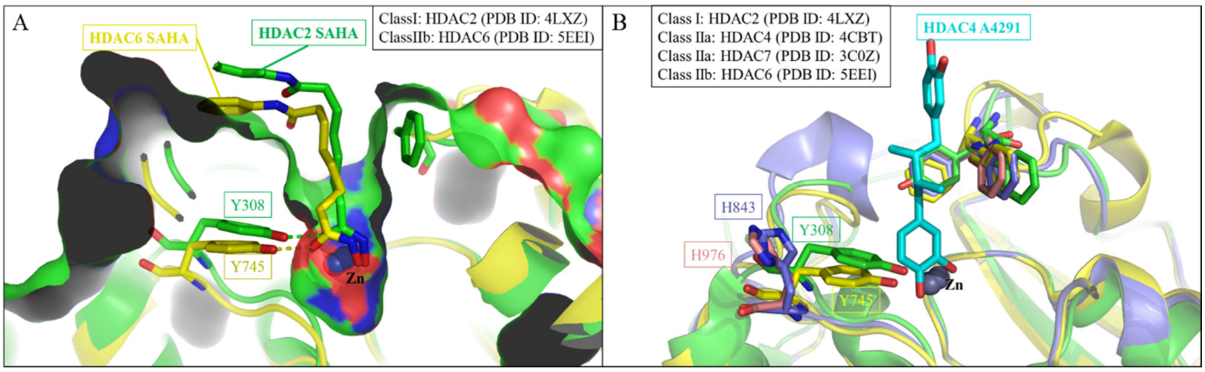

Supplementary Figure 7. SAHA interactions in HDAC isozymes.

(A) HDAC2 (green) and HDAC6 (yellow) complex was aligned and a surface model was produced. The tyrosine residue is labeled as shown. (B) The structures of class I (HDAC2, green), IIa (HDAC4, pink; HDAC7, blue), and IIb (HDAC6, yellow) are aligned. Inhibitor A4291, posed from HDAC4, is aligned within the active site. The grey sphere represents the zinc ion.

A

Compound	Ranking		
	DS	LeadIT	iGEMDOCK
NSC159452	33	31	13
NSC226640	15	8	18
NSC363007	1	1	1
NSC364365	27	5	11
NSC4291	14	16	24
NSC640973	23	2	10

B

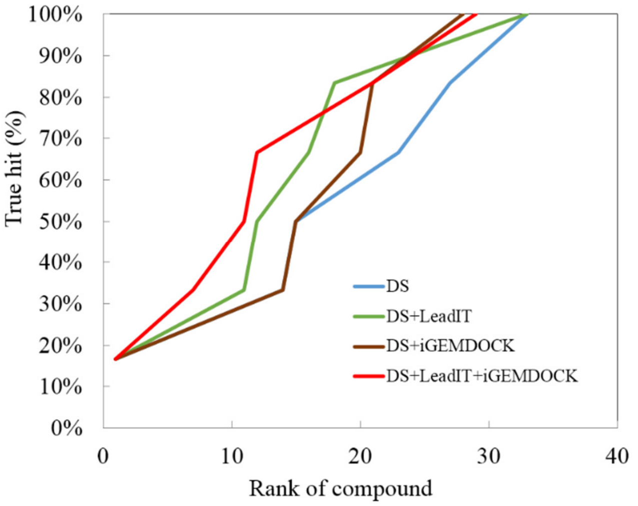

Supplementary Figure 8. Ranking and hit rate of docking software.

(A) Ranking order of the identified inhibitors across three programs. (B) Hit rate of different methods. The six inhibitors are docked and ranked in DS, LeadIT or iGEMDOCK. The hit rate is defined as *I*/*T* (%), where *I* is the number of the identified inhibitors among the *T* highest-ranking compounds The consensus score (red line) improves the ranking of the inhibitors against 27 false positives.
